# HPV Vaccination in Young Males: A Glimpse of Coverage, Parental Attitude and Need of Additional Information from Lombardy Region, Italy

**DOI:** 10.3390/ijerph19137763

**Published:** 2022-06-24

**Authors:** Alessandra Mari, Laura Gianolio, Valeria Edefonti, Dariush Khaleghi Hashemian, Francesca Casini, Francesco Bergamaschi, Anna Sala, Elvira Verduci, Valeria Calcaterra, Gian Vincenzo Zuccotti, Valentina Fabiano

**Affiliations:** 1 Department of Pediatrics, V. Buzzi Children’s Hospital, Università degli Studi di Milano, 20154 Milano, Italy; alessandra.mari@unimi.it (A.M.); laura.gianolio@unimi.it (L.G.); francesca.casini@unimi.it (F.C.); francesco.bergamaschi@unimi.it (F.B.); anna.sala1@unimi.it (A.S.); elvira.verduci@unimi.it (E.V.); valeria.calcaterra@unipv.it (V.C.); gianvincenzo.zuccotti@unimi.it (G.V.Z.); 2 Laboratory of Medical Statistics, Biometry and Epidemiology “G. A. Maccacaro”, Department of Clinical Sciences and Community Health, Università degli Studi di Milano, 20133 Milano, Italy; valeria.edefonti@unimi.it (V.E.); dariush.khaleghi@unimi.it (D.K.H.); 3 Fondazione IRCCS Ca’ Granda Ospedale Maggiore Policlinico, 20122 Milano, Italy; 4 Department of Health Sciences, Università degli Studi di Milano, 20146 Milano, Italy; 5 Department of Internal Medicine and Therapeutics, University of Pavia, 27100 Pavia, Italy; 6 Department of Biomedical and Clinical Sciences, Università degli Studi di Milano, 20147 Milano, Italy

**Keywords:** HPV, vaccination, males, pediatric practice, knowledge and attitude

## Abstract

**Background:** In the Lombardy Region, Italy, HPV vaccination is recommended and offered free of charge to 12-years-old males since 2017. The expected vaccination thresholds are still far to be reached. **Methods:** A cross-sectional survey to investigate parents’ attitudes towards the HPV vaccine and knowledge about HPV was administered to parents of boys aged 6 to 18 years attending a large pediatric hospital for outpatient specialistic evaluations. Two parallel multiple logistic regression analyses were conducted to estimate the odds ratios (ORs) and the corresponding 95% confidence intervals (CIs) for attitude towards HPV vaccination and perceived need for more information about HPV vaccination. **Results:** A positive attitude towards HPV vaccination was found in 74% of interviewed parents. Knowledge of HPV, having a generally positive attitude toward vaccination, and mothers filling in the survey were positively associated with a positive attitude to the HPV vaccine. Parents’ perceived need for more information about HPV vaccination was positively associated with the child’s age, general positive attitude toward vaccination, Christian religion, and positive attitude toward HPV vaccination; knowing that HPV vaccination is free of charge significantly reduced the risk of asking for more information on HPV vaccination. **Conclusions:** The majority of parents of male children and adolescents in our study have a positive attitude toward HPV vaccination. Attitude toward HPV vaccination and perceived need for more information on HPV vaccination were directly related to a positive attitude toward vaccines in general. In addition, knowledge of HPV and related pathologies favors a positive attitude toward HPV vaccination. Future health programs should target an even wider diffusion of evidence-based information on vaccines in general and on the HPV vaccine in young males, to support a positive attitude toward vaccines in the general population.

## 1. Introduction

Human Papillomavirus (HPV) infection is the most common sexually transmitted infection worldwide, as up to 80% of sexually active men and women are expected to encounter it at least once in their lifetime [1,2]. Cervical cancer, the fourth most common malignant tumor in women, with an incidence of 8.4/100.000 women/year in high-income countries [3], has virtually no other etiology than persistent HPV infection sustained by high-risk genotypes [4,5]. The burden of HPV-related diseases affects men too, as high-risk HPV genotypes are also associated with oropharyngeal, penile, and anal cancer [6,7], whose incidences, in high-income countries, have been estimated at 3.28, 0.66, and 0.74/100.000 men/year, respectively [3]. The most comprehensive systematic review on males—conducted in 2014—found a prevalence of HPV among European males of 12.5% in the general population and 30.9% in high-risk groups [8]. Focusing on the Italian situation, the incidence of cervical cancer in women is estimated to be 6.9/100.000/year [3]; 4.1% of women in the general population have been estimated to harbor cervical HPV-16/18 infection, and 72.2% of invasive cervical cancers have been attributed to HPVs 16 or 18; in men, a higher prevalence of external genital HPV infection has been described, varying from 8.7 to 40.5%, although HPV persistence is less likely [3]. Due to HPV diffusion and its potential morbidity, great efforts have been made in prevention strategies.

Prevention strategies for HPV-related diseases still comprise screening programs for women based on cytological smears; in addition to this, over the years, different HPV vaccines have been developed. Currently, there are three prophylactic HPV vaccines available (bivalent, quadrivalent, and nonvalent). All of them target the high-risk HPV genotypes (HPV-16 and HPV-18) plus other low-risk genotypes (HPV-6 and HPV-11 for the 4-valent, and HPV-6, HPV-11, HPV-31, HPV-33, HPV-45, HPV-52, and HPV 58 for the 9-valent vaccine, respectively), which are associated to anogenital warts and low-grade abnormalities. Previous studies have shown the vaccines’ efficacy in reducing the transmission and prevalence of HPV infection and cervical abnormalities [9,10,11].

In 2007, Italy began its campaign for immunization recommending the HPV vaccine to girls aged 11–12 years and offering it free of charge. With the latest National Vaccination Prevention Plan (PNPV), issued in 2017, the offer was extended to boys of the same age, starting from the 2006 cohort [12]. The PNPV had set the goal of reaching >95% of vaccination coverage for HPV in both girls and boys eligible to receive the vaccine by the year 2020. Presently, this goal is far from being reached. According to the latest report available, in 2020 only 30.3% of Italian females of the 2008 cohort and 24.2% of males of the same cohort received a complete course of HPV vaccination, with inter-regional differences (range 6–53.9%% for girls and 5.4–46.8% for boys). In Lombardy Region, in the same 2008 cohort, only 17.7% of females and 16.5% of males had received a complete course of HPV vaccination [13].

Previous studies have investigated the knowledge of HPV and attitude toward HPV vaccination in adolescent or adult men [14,15,16,17,18], healthcare providers [19,20], and parents of boys [21,22,23,24,25]. In 2013, before the introduction of free of charge HPV vaccination, a first local survey on HPV vaccination has been conducted among parents of adolescent boys, in northern Italy. The survey, conducted in three municipalities of Brescia province in the Lombardy Region, demonstrated a largely positive attitude of parents of males toward HPV vaccination; 116 questionnaires, with a 15% response rate, were completed and 97% of interviewed parents declared to be willing to vaccinate their sons [26]. To our knowledge, this is the first Italian study specifically addressing parents of boys, after the introduction of free of charge HPV vaccination in males.

Considering the HPV-infection burden in men and women together with the increased availability of effective and safe vaccination strategies supported by the latest PNPV, we aimed to investigate the factors potentially predicting the unsatisfactory HPV vaccination coverage in Italian boys observed so far. In detail, we will examine either attitude toward HPV vaccination or the perceived need for more information on HPV, as well as their determinants, in parents of boys interviewed in a large hospital in the greater Milan area, in the Lombardy region, Italy. While shedding light on the gap between expected and actual coverage, we expect to inform future strategies in support of vaccination adherence.

## 2. Materials and Methods

### 2.1. Setting and Data Collection

Upon approval from the Ethical Committee of the University of Milan, we conducted an anonymous survey regarding HPV vaccination in boys from November 2021 to February 2022. Eligible subjects were parents of boys aged 6–18 years who attended the large pediatric Buzzi hospital in Milan for outpatient specialistic visits in the study period. We excluded those subjects who were not able to read and understand the questionnaire in one of the proposed languages. After informed consent was signed, a questionnaire (details in the following) was delivered to one parent/caregiver and filled in in the waiting room. Trained medical personnel were available for any upcoming question. Upon completion, parents received a leaflet with information about HPV infection and vaccination.

### 2.2. Questionnaire

We specifically developed a 15-item self-administered questionnaire to evaluate parents’ knowledge and acceptability of the HPV vaccine in boys, based on an extensive literature search of factors related to vaccine acceptability, as well as questionnaires used in similar studies [20,21,22,26,27,28]. The first version of the questionnaire was pilot-tested on 15 Italian parents and adjusted following their feedback. The questionnaire was further translated by certified resident cultural mediators, who are usually available at Buzzi Hospital during working days and have broad experience in linguistic and cultural mediation, including translation of medical and scientific documents, into English, Spanish, Arabic, and Chinese languages for favoring a broader evaluation of vaccination attitudes in all parents residing in Italy.

After socio-demographic characteristics of parents and children were collected, the core focus of the questionnaire assessed parents’ knowledge about HPV infection and availability of free-of-charge vaccines for males and females, parents’ general attitude toward vaccines, and their attitude toward HPV vaccination for their son. Reasons for not vaccinating were also investigated. Finally, the last two questions addressed parents of boys >15 years who had not been included in the PNPV and were not offered the HPV vaccine for free. The administered questionnaires (in all different languages used) are available in the Supplementary Materials.

### 2.3. Sample Size

The sample size was calculated considering a Chi-square test of independence between the three-level “attitude toward HPV vaccination” variable and any other possible three-level categorical variable of interest. Assuming an alpha value of 0.05 and a power of 0.80, the number of responders needed to reach an effect size of 0.20 with 4 degrees of freedom was 299. Accounting for a potential dropout of 20%, the final sample size to collect was set at 360.

Subjects were included in the study until the expected sample size of 360 parents was reached. A total of 27 participants were excluded from the analysis due to incomplete questionnaires. A total sample size of 339 parents was considered in the final analysis.

### 2.4. Statistical Analysis

The study population was initially characterized through descriptive statistics, including absolute frequencies and percentages for categorical variables and mean and standard deviation for continuous variables because the corresponding distributions were symmetric.

Two parallel multiple logistic regression analyses were further conducted to estimate the odds ratios (ORs) and the corresponding 95% confidence intervals (CIs) for each of the following outcome variables: attitude towards HPV vaccination (dichotomized as: yes/already vaccinated vs. no) and perceived need of more information about HPV vaccination (yes vs. no). A confounding-factor-only model was initially fitted that included a selection of five confounding factors derived from a careful examination of the literature. The final regression model was obtained by taking the confounding-factor-only model for granted and adding the best set of all the remaining variables with a stepwise selection approach.

We also examined pairwise interaction terms in the confounding-factor-only model and in the final model. In detail, we added one interaction term at a time to the main-effects model; evidence of a potential interaction effect was explored with the likelihood ratio test comparing main-effects models including versus excluding each interaction term.

Significance was assumed when *p* < 0.05 for the main effects and when *p* < 0.1 for the interaction effects. The statistical analysis was performed using the R software, version 4.1.2 (1 November 2021).

## 3. Results

Socio-demographic characteristics of the sample are reported in Table 1. In most cases, questionnaires were filled in by mothers, interviewed parents were Italian and of the Christian religion. The mean age of the boys and parents was 11.5 and 44.6 years, respectively. In our sample, parents had a generally high education level, with only 6.2% who did not reach a high school diploma.

Table 2 summarizes knowledge and perceived need for more information on the HPV vaccine, as well as attitudes toward vaccination (in general and for HPV) in our sample. Even though 56.9% of the parents stated that they knew HPV and related diseases, and 20.1% had at least heard about it, 63.4% would like to have more information about it. For a third of the parents (34.8%), the main source of information was the Pediatrician. Almost half of the parents (44.8%) were unaware that the HPV vaccine is offered free of charge to 12 years old boys in Lombardy.

Here, 90% of parents had a positive attitude toward vaccines in general vs. 74% had a positive attitude toward HPV vaccination. Among the latter, 20.6% of parents had their son already vaccinated and 53.4% of parents were willing to have him vaccinated. Almost half of those who did not want to vaccinate their sons indicated the lack of information as the main motivation.

Among boys older than 15 years—to whom HPV vaccine was not offered for free when they were 12 years old—only 27.7% were vaccinated, whereas 61.7% of parents whose sons were not vaccinated would have done it if it had been free of charge.

Table 3 shows raw associations between available confounding/risk factors and attitude toward HPV vaccination (left part of the table) and the perceived need for more information on HPV vaccine (right part of the table).

Table 4 shows the ORs of a positive attitude toward HPV vaccination and corresponding 95% CIs for selected confounding factors. In the absence of significant two-way interactions, the confounding-factor-only model and the final model included only main effects. The confounding-factor-only model showed significant effects of parental age and source of information on positive attitudes toward HPV vaccination. In detail, younger parents were less likely (~60% less favorable) to vaccine their sons against HPV (OR = 0.37, 95% CI: 0.15–0.93); when information on HPV vaccine came from HCP, parents were more likely (~5 higher risk) to vaccinate their sons against HPV (OR = 4.50, 95% CI: 2.55–7.93). However, these associations disappeared in the final model including variables from the stepwise-based model selection. In detail, each of the following variables: parent who filled in the questionnaire, knowledge about HPV, and general attitude toward vaccination, was positively and significantly related to attitude toward HPV vaccination. A general attitude toward vaccination led to a 3.5 higher “risk” of son vaccination (OR = 3.47, 95% CI: 1.42–8.50). Having some knowledge about HPV led to a 2.5 higher “risk” of son vaccination (OR = 2.49, 95% CI: 1.22–5.11). When the mother filled in the questionnaire, her son doubled his “risk” of being vaccinated (OR = 2.27, 95% CI: 1.21–4.26). In addition, knowing that vaccine is free led to a borderline significant OR of 2.05 (95% CI: 1.00–4.20), which means that sons of aware parents still doubled their “risk” of being vaccinated.

Table 5 shows the ORs of a perceived need for more information about the HPV vaccine and corresponding 95% CIs for selected confounding factors. In the absence of significant two-way interactions, the confounding-factor-only model and the final model included only main effects. In the confounding-factor-only model, parents of sons younger than 12 years more than doubled their “risk” of asking for more information about the HPV vaccine (OR = 2.45, 95% CI: 1.43–4.22). A positive attitude toward the HPV vaccine provided a ~2 higher risk of requiring HPV vaccine information (OR = 2.14, 95% CI: 1.17–3.93). On the contrary, receiving information from HCP and having a son already vaccinated were inversely and significantly related to the “risk” of asking for more information about the HPV vaccine. In detail, the “risk” of asking more information about the HPV vaccine was halved, compared to those parents who received information from family/friends or other sources (OR = 0.55, 95% CI: 0.33–0.94) and for those who had already vaccinated another son (OR = 0.41, 95% CI: 0.19–0.89). In the final model including selected confounding factors, the associations with the outcome were similar for having a son younger than 12 years and having a positive attitude toward the HPV vaccine. On the contrary, significance was lost for information from HCP and having already vaccinated another son, although the ORs were in the same direction as those from the confounding-factor-only model. In addition, the final model also included other three variables significantly related to the “risk” of asking for more information about the HPV vaccine. A general attitude toward vaccination more than tripled the “risk” of asking for more information (OR = 3.62, 95% CI: 1.51–8.70), whereas being Christian almost doubled this “risk” (OR = 1.91, 95% CI: 1.04–3.50). On the other hand, having already known that the HPV vaccine is free led to a reduced “risk” of asking for additional information with an OR equal to 0.27 (95% CI: 0.14–0.53).

## 4. Discussion

HPV infection is a cause of significant medical burden in both female and male populations, representing a public health priority. Therefore, vaccine-based preventive strategies have been increasingly available worldwide with target population extending toward young boys, as reported in the latest PNPV. Despite these measures, the goal of reaching >95% of HPV vaccination coverage in both girls and boys is far from being achieved.

Factors underlying reduced parental compliance toward HPV vaccination have been investigated by previous studies, which, however, are often not concordant in their results. Most of the current evidence derives from studies carried out among parents of females, and the majority of them were not Italian studies. Our study is the first to be conducted in Italy, specifically addressing parents of boys, after the introduction of free of charge HPV vaccination in males.

Our study reports that, overall, 74% of interviewed parents reported having a positive attitude toward HPV vaccination: 53.4% reported being willing to have their sons vaccinated and 20.6% reported having already vaccinated them. Previously published papers, though conducted in northern America and Asia, found a similar percentage of boys’ parents willing to vaccinate, ranging from 45% to 67% [21,23,25,29,30].

Considering parents’ attitudes toward HPV vaccination, the major influencing factors were a positive attitude toward vaccines in general and a reported knowledge of HPV and related pathologies. These findings are consistent with previous evidence, both in males’ and females’ parents [22,23,25,31,32,33]. Moreover, similarly to what had been previously reported [23,34], parents who reported to have been informed about HPV infection and HPV vaccine by HCP were more likely to agree to vaccinate their sons rather than those reporting to have been informed by other sources of information; however, significance was lost after adjustment for other confounding factors. Focusing on parents reporting to be against HPV vaccination, almost half indicated the lack of information as the main motivation; a similar observation has been made by other authors [35]. A large national survey, conducted among US parents, has demonstrated that addressing the lack of knowledge about HPV vaccination and giving messages that include information about the prevention of cancer are associated with increased parental confidence in HPV vaccination [36]. Our findings support this theory and highlight once again the importance of extensive information campaigns as a strategy to achieve the desired immunity thresholds and reinforce the need to increase physicians’ communication with parents of boys regarding the HPV vaccine, considering the greater value recommendations assume when given by Pediatricians or HCP.

In accordance with Nguyen’s recent findings [25], in our study, mothers were more likely than fathers to agree to have their son vaccinated. This observation is in line with another study in which mothers have been identified as the primary decision-makers regarding childhood vaccinations [37]. On the contrary, other older studies did not find such an association [21,24].

A systematic review published in 2014 identified vaccine cost as a barrier to against HPV vaccination in children [34]. In a Chinese study, acceptability of HPV vaccination was price-sensitive for both boys and girls, with an inverse association between vaccine cost and rate of willingness to vaccinate; however, parents of boys reported significantly lower acceptability of HPV vaccine than those of girls, regardless of vaccine cost [38]. In our study, the knowledge that the Lombardy region offers HPV vaccination to 12 years-old adolescents for free positively influences parents’ willingness to have their sons vaccinated, but the OR is of borderline significance. As expected, a free HPV vaccination is appreciated by parents. Indeed, the OR in the univariate model including knowledge of free vaccination only was 4.94 (95% CI: 2.89–8.44) (data not shown). However, when major confounding and risk factors were entered into the multiple models, the CI included unity (OR *=* 2.05, 95% CI: 1.00–4.20). In the absence of significant interactions involving knowledge of free HPV vaccination, this suggests that free of charge vaccination is not as important for parents in their decision-making as are other factors.

Parental education did not influence vaccination acceptability in our study. However, results from the literature are mixed, with some older studies reporting an inverse association [22,28,31,32,39,40,41], and other recent studies suggesting a positive one [25,42]. Based on our findings, we may speculate that our population has already reached a good awareness of the importance of HPV vaccination in adolescents, especially males, independently of their education level. This might suggest that some important results have been already achieved in the breakdown of cultural and social barriers to vaccinations’ knowledge and uptake, which may be associated with healthcare access disparities. This finding is even more important considering that cancer and other HPV-related diseases tend to disproportionally affect low income and low education minorities [43].

As in previous studies, we did not find strong associations between demographic characteristics and vaccination uptake in males [24]. Parental age was modestly and inversely related to willingness to vaccinate but significance was lost in the final model. The age of the boys and parents’ nationality and religion were not significant predictors of vaccine uptake. On the contrary, other authors reported stronger associations with some specific demographic factors, such as ethnicity [40,44,45]. Similarly, results on the effect of religion are mixed, with some authors suggesting religious parents have positive beliefs about the HPV vaccine [46], and others reporting a lower HPV vaccine acceptance in parents with a religious belief [47]. In accordance with our findings, two studies performed in Canada and Sweden did not find any influence of religion on HPV vaccine uptake and consent [22,48]; similarly to our study, parents in the Canadian study mostly declared being Christian or having no specific religious belief.

As knowledge about HPV strongly influences parents’ willingness to vaccinate their sons, we further focused on the perceived need for information about the HPV vaccine and its determinants, a less investigated topic in all the previously cited papers. A child’s age was significantly and inversely related to parents’ perceived need for information. This suggests awareness-raising campaigns and informative conversations with pediatricians may be targeted at parents of younger children, thus encouraging earlier and better parents’ acceptability toward HPV vaccination.

Unsurprisingly, parents reporting a positive attitude toward vaccines in general and parents reporting the intention to have their sons vaccinated were also more interested in receiving information about the HPV vaccine. In our study, parents reporting already know that vaccine is free of charge were less prone to be informed, because they have been likely in contact with someone who provided some information on HPV vaccination.

On the contrary, the parental perceived need for information was not influenced by their age, nationality, and education level, suggesting a limited impact of demographic factors. Religion was, however, an exception: contrary to its null effect on parents’ willingness to vaccinate, being Christian was positively and significantly associated with a perceived need for more information about the HPV vaccine.

Our study has some limitations. First, as an intrinsic limitation of survey-based research, our results may have been affected by response bias. The questionnaire was completed by parents while waiting for an outpatient specialistic evaluation for their sons in the hospital waiting room, thus, their participation in the study may have been influenced both by the setting and by a possible pre-existing greater attention to health care-related behaviors. Secondly, different attitudes related to religious beliefs may have been underestimated, since the catholic religion is still the most largely professed one in our sample. Third, our survey has been conducted during the third wave of the COVID-19 pandemic and while starting the COVID-19 immunization campaign for 5–11-year-old children in Italy. Thus, we cannot exclude that a generally better attitude toward vaccinations during the pandemic time may have influenced either survey participation or our findings on HPV vaccination attitude.

Our study has also some strengths. The expected sample size was reached, and this is especially worthy note if compared to the relatively short recruitment window. This is due in part to the availability of several outpatient ambulatories within V. Buzzi Children’s Hospital. In addition, this hospital is one of the two large pediatric reference centers available in Milan. Its catchment area includes also patients belonging to the greater Milan area, as it represents a point of reference for several pediatric specialties. Our sample is multiethnic, as nearly 15% of interviewed parents were foreigners, a higher percentage than the one registered in Italy and Lombardy Region (8.8% and 11.9%, respectively) [49]. Therefore, our study population may be considered quite representative of the multiethnicity of the population living in Italy and, specifically, in the Lombardy Region. Our research has been conducted after the PNPV recommendation to vaccinate boys and the introduction of a free-of-charge HPV vaccine for males; thus, it may better capture the parental attitude toward HPV vaccination as compared to earlier surveys, when HPV immunization in male adolescents was still not recommended.

## 5. Conclusions

Our results show that the majority of parents of male children and adolescents have a positive attitude toward HPV vaccination. It represents a limited but positive signal, even if HPV vaccine coverage is still far from meeting the 2017 PNPV goals. Attitude toward HPV vaccination and perceived need for more information on HPV vaccination were directly related to a positive attitude towards vaccines in general. In addition, knowledge of HPV and related pathologies favor a positive attitude toward HPV vaccination. Similarly, lack of information was indicated, in our study, to be the most common reason for parents to refuse vaccination. Future health programs should target an even wider diffusion of evidence-based information on vaccines in general and, specifically, on primary prevention of cancer and other HPV-related diseases through vaccination in both girls and boys. This effort will itself provide support to a stronger positive attitude toward vaccines in the general population.

## Figures and Tables

**Table 1:** Socio-demographic characteristics of the population.

Characteristic	*n* = 339
**Age of sons (years)**	11.5 ± 3.2 *
**Age of parents (years)**	44.5 ± 6.2 *
**Mother filled in survey**	258 (76.1%)
**Nationality of the parent**	
Italian	289 (85.2%)
Other	50 (14.8%)
**Religion of the parent**	
Christian	255 (75.5%)
Atheist	55 (16.2%)
Muslim	24 (7.1%)
Other	5 (1.5%)
**Education of parent**	
Lower than high school diploma	21 (6.2%)
High school diploma	164 (48.4%)
College degree	154 (45.4%)

* Mean ± standard deviation.

**Table 2:** HPV infection and vaccine knowledge.

HPV Knowledge and Vaccine Acceptability	*n* = 339
**Know HPV and the related pathologies**	
Yes	193 (56.9%)
Heard about it	68 (20.1%)
No	78 (23.0%)
**Source of information**	
Pediatrician	118 (34.8%)
Friends and Family	90 (26.5%)
Vaccination Center	53 (15.6%)
Other	78 (23.1%)
**Would like more information on HPV**	
Yes	215 (63.4%)
No	124 (36.6%)
**Know that HPV vaccine is free**	
Yes	187 (55.2%)
No	152 (44.8%)
**General attitude toward vaccines**	
Positive	305 (90.0%)
Negative	11 (3.2%)
Doesn’t know	23 (6.8%)
**Attitude toward HPV vaccination**	
Already vaccinated	70 (20.6%)
Positive	181 (53.4%)
Negative	88 (26%)
**Reasons to not vaccinate**	*n* = 88
Lack of information	42 (47.7%)
Choice	18 (20.5%)
Not reported	28 (31.8%)
**Son > 15 years**	*n* = 65
Vaccinated	18 (27.7%)
Unvaccinated	47 (72.3%)
**Would have vaccinated with free HPV vaccine**	*n* = 47
Yes	29 (61.7%)
No	18 (38.3%)

**Table 3:** Distribution of attitude toward HPV vaccination or perceived need of more information on HPV vaccine according to selected variables.

	Attitude toward HPV Vaccination ^a^—Negative (%)		Attitude toward HPV Vaccination ^a^—Positive (%)		Perceived Need of More Information on HPV Vaccine ^a^—No (%)		Perceived Need of More Information on HPV Vaccine ^a^—Yes (%)	
**Age of son (years)**								
**≥12**	39	(44.3)	135	(53.8)	90	(72.6)	84	(39.1)
**<12**	49	(55.7)	116	(46.2)	34	(27.4)	131	(60.9)
**Age of parent (years)**								
**>35**	76	(86.4)	239	(95.2)	119	(96)	196	(91.2)
**≤** **35**	12	(13.6)	12	(4.8)	5	(4)	19	(8.8)
**Nationality**								
Other	18	(20.5)	32	(12.7)	14	(11.3)	36	(16.7)
Italian	70	(79.5)	219	(87.3)	110	(88.7)	179	(83.3)
**Religion**								
Other/Atheist	29	(33)	55	(21.9)	36	(29)	48	(22.3)
Christian	59	(67)	196	(78.1)	88	(71)	167	(77.7)
**Education level**								
Lower than college degree	56	(63.6)	129	(51.4)	68	(54.8)	117	(54.4)
College degree	32	(36.4)	122	(48.6)	56	(45.2)	98	(45.6)
**Filled in by**								
Father	35	(39.8)	46	(18.3)	25	(20.2)	56	(26)
Mother	53	(60.2)	205	(81.7)	99	(79.8)	159	(74)
**HPV Knowledge**								
No	44	(50)	34	(13.5)	22	(17.7)	56	(26)
Yes	44	(50)	217	(86.5)	102	(82.3)	159	(74)
**Source of knowledge**								
Other	66	(75)	102	(40.6)	46	(37.1)	122	(56.7)
HCP	22	(25)	149	(59.4)	78	(62.9)	93	(43.3)
**Knowledge of free HPV vaccine**								
No	64	(72.7)	88	(35.1)	31	(25)	121	(56.3)
Yes	24	(27.3)	163	(64.9)	93	(75)	94	(43.7)
**Perceived need of more information on HPV vaccinea**								
No	31	(35.2)	93	(37.1)	-		-	
Yes	57	(64.8)	158	(62.9)	-		-	
**Attitude toward HPV vaccination ^a^**								
Negative	-		-		31	(25)	57	(26.5)
Positive	-		-		93	(75)	158	(73.5)
**Other children already vaccinated**								
No	81	(92)	189	(75.3)	98	(79)	172	(80)
Yes	7	(8)	62	(24.7)	26	(21)	43	(20)
**General attitude toward vaccination**								
No	23	(26.1)	11	(4.4)	18	(14.5)	16	(7.4)
Yes	65	(73.9)	240	(95.6)	106	(85.5)	199	(92.6)

HCP (Health care providers), NA (not applicable). ^a^ We cross-tabulated attitudes toward HPV vaccination and perceived need for more information on the HPV vaccine. Corresponding frequencies were the same in both subtables, but they were just switched. No table was available when the same variable was presented in rows and columns. For “Attitude toward HPV vaccination”, the positive category here included also those parents who had already vaccinated their son against HPV.

**Table 4:** Odds ratios (ORs) for a positive attitude toward HPV vaccination and corresponding 95% confidence intervals (CIs) for selected confounding and risk factors.

	Confounding-Factor-Only Model ^a^			Final Model ^b^		
	OR	95% CI		OR	95% CI	
**Age of children**						
≥12 years	1 ^c^			1 ^c^		
<12 years	0.78	0.44	1.36	0.80	0.43	1.47
**Age of parent**						
>35 years	1 ^c^			1 ^c^		
≤35 years	**0.37** ^d^	**0.15**	**0.93**	0.51	0.19	1.42
**Education level of parent**						
Under college graduation	1 ^c^			1 ^c^		
College graduate	1.42	0.83	2.44	1.08	0.60	1.95
**Source of knowledge**						
Other	1 ^c^			1 ^c^		
HCP	**4.50**	**2.55**	**7.93**	1.52	0.74	3.14
**Perceived need of more information on HPV vaccine**						
No	1 ^c^			1 ^c^		
Yes	1.44	0.80	2.58	1.50	0.76	2.99
**Filled in by**						
Father				1 ^c^		
Mother				**2.27**	**1.21**	**4.26**
**Knowledge of HPV**						
No				1 ^c^		
Yes				**2.49**	**1.22**	**5.11**
**Knowledge of free HPV vaccine**						
No				1 ^c^		
Yes				2.05	1.00	4.20
**Other children vaccinated**						
No				1 ^c^		
Yes				1.99	0.82	4.85
**General attitude toward vaccines**						
Negative				1 ^c^		
Positive				**3.47**	**1.42**	**8.50**

HCP (health care providers). ^a^ Estimated from multiple logistic regression models adjusted for confounding factors selected based on the literature. ^b^ Estimated from multiple logistic regression models adjusted for confounding factors selected based on the literature and additional risk factors obtained from automatic model selection. ^c^ Reference category. ^d^ The odds ratios whose confidence intervals do not include unity are indicated in bold typeface.

**Table 5:** Odds ratios (ORs) for perceived need of more information on the HPV vaccine and corresponding 95% confidence intervals (CIs) for selected confounding and risk factors.

	Confounding-Factor-Only Model ^a^			Final Model ^b^		
	OR	95% CI		OR	95% CI	
**Age of children**						
≥12 years	1 ^c^			1 ^c^		
<12 years	**2.45 ^d^**	**1.43**	**4.22**	**2.23**	**1.26**	**3.94**
**Age of parents**						
>35 years	1 ^c^			1 ^c^		
≤35 years	1.44	0.47	4.36	1.09	0.33	3.58
**Education level of parent**						
Under college graduation	1 ^c^			1 ^c^		
College graduate	0.98	0.59	1.65	1.05	0.61	1.81
**Source of knowledge**						
Other	1 ^c^			1 ^c^		
HCP	**0.55**	**0.33**	**0.94**	0.72	0.39	1.33
**Attitude toward HPV vaccine**						
Negative	1 ^c^			1 ^c^		
YesPositive	**2.14**	**1.17**	**3.93**	**2.15**	**1.08**	**4.26**
Already vaccinated	**0.41**	**0.19**	**0.89**	0.44	0.19	1.04
**Religion**						
Other				1 ^c^		
Christian				**1.91**	**1.04**	**3.5**
**Knowledge of free HPV vaccine**						
No				1 ^c^		
Yes				**0.27**	**0.14**	**0.53**
**General attitude toward vaccines**						
Negative				1 ^c^		
Positive				**3.62**	**1.51**	**8.7**

HCP (health care providers). ^a^ Estimated from multiple logistic regression models adjusted for confounding factors selected based on the literature. ^b^ Estimated from multiple logistic regression models adjusted for confounding factors selected based on the literature and additional risk factors obtained from automatic model selection. ^c^ Reference category. ^d^ The odds ratios whose confidence intervals do not include unity are indicated in bold typeface.

## Data Availability

Data supporting the results of this study can be obtained, upon reasonable request, from the corresponding author (V.F.).

## Supplementary Materials

The original questionnaire and the English, Arabic, Chinese and Spanish translations can be downloaded at: https://www.mdpi.com/article/10.3390/ijerph19137763/s1.
