# Supplementary material for: HPV Vaccination in Young Males: A Glimpse of Coverage, Parental Attitude and Need of Additional Information from Lombardy Region, Italy
Source: Int J Environ Res Public Health. 2022 Jun 24;19(13):7763. doi: 10.3390/ijerph19137763 (PMC9265455; doi:10.3390/ijerph19137763)
Supplement: Supplementary file 1 [file ijerph-19-07763-s001.zip › Questionnaire HPV English.pdf]

HPV-VACCINATION AWARENESS AND ACCETTABILITY: a survey among parents of male children and adolescents

Child's age:……………….. **Parent's age**:……………

Parent's nationality:……………….. **Parent's religion**: ………………..

Survey filled in by: [ ] Father [ ] Mother

Parent's level of education: [ ] Middle school diploma [ ] High school diploma [ ] College degree

1) **Are you aware of the existence of HPV viruses and of the pathologies associated to them?**

[ ] Yes [ ] I've heard about them [ ] No

2) **If yes, from whom have you heard about it?**

[ ] Pediatrician [ ] Vaccination Centre [ ] Friends/Relatives

3) **Do you know that Regione Lombardia offers HPV vaccination for free to all males born after 2006?**

[ ] YES [ ] NO

4) **Would you like to have more information about it?**

[ ] YES [ ] NO

5) **Are you planning on having your son vaccinated for HPV?**

[ ] He is already vaccinated [ ] YES [ ] NO

If not, why? ……………………………………………………………………

6) **Do you have any other child who is vaccinated for HPV? (If yes, please specify sex and age)**

[ ] NO [ ] YES …………………………………………………

→ → → → → → → **TURN THE PAGE!**→ → → → → → →

7) **Generally speaking, do you think vaccinations are a valid help to prevent infectious diseases?**

[ ] YES [ ] NO [ ] Uncertain

For parents of children older than 15 years:

a) Is your son vaccinated for HPV?

[ ] YES [ ] NO

b) If the HPV vaccine had been free of charge for your son, would you have had him vaccinated?

[ ] YES [ ] NO
