# Supplementary material for: HPV Vaccination in Young Males: A Glimpse of Coverage, Parental Attitude and Need of Additional Information from Lombardy Region, Italy
Source: Int J Environ Res Public Health. 2022 Jun 24;19(13):7763. doi: 10.3390/ijerph19137763 (PMC9265455; doi:10.3390/ijerph19137763)
Supplement: Supplementary file 1 [file ijerph-19-07763-s001.zip › Questionnaire HPV Spanish.pdf]

ENCUESTA DE CONOCIMIENTO SOBRE LA VACUNACIÓN CONTRA EL VPH

Cuestionario dirigido a los padres de niños y adolescentes varones

Edad del niño:……………….. **Edad del padre o madre**:……………

Nacionalidad del padre o madre:………………..

Religión del padre o madre: ………………..

Cuestionario rellenado por: [ ] Padre [ ] Madre

Estudios alcanzados por el padre o madre:

[ ] ESO (Escuela Secundaria Obligatoria) [ ] Bachiller [ ] Titulo universitario

1) ¿Conoces el virus VPH y sabes a qué patologías está asociado?

[ ] Si [ ] Solo he oído hablar [ ] No

2) **En caso afirmativo, ¿quién se lo dijo?**

[ ] Pediatra [ ] Centro de vacunación [ ] Amigos/Familiares

3) **¿Sabías que en la región de Lombardía la vacuna se ofrece gratuitamente a los varones nacidos a partir de 2006?**

[ ] SI [ ] NO

4) **¿Le gustaría tener más información al respecto?**

[ ] SI [ ] NO

5) ¿Va a vacunar a su hijo?

[ ] Ya vacunado [ ] SI [ ] NO

En caso negativo, ¿por qué?
……………………………………………………………………

→ → → → → → → ¡SIGUE POR LA OTRA CARA! → → → → → → →

6) **¿Tienes otros hijos/hijas vacunados contra el VPH? (si la respuesta es afirmativa, especifique el sexo y la edad)**

[ ] NO [ ] SI ....

7) **En general, ¿está usted a favor de las vacunas?**

[ ] SI [ ] NO [ ] No lo sé

Para padres de niños de más de 15 años:

a) ¿Está su hijo vacunado contra el VPH?

[ ] SI [ ] NO

b) Si la vacuna hubiera estado entre las vacunas ofrecidas gratuitamente, ¿habría vacunado a su hijo?

[ ] SI [ ] NO
