# Supplementary material for: HPV Vaccination in Young Males: A Glimpse of Coverage, Parental Attitude and Need of Additional Information from Lombardy Region, Italy
Source: Int J Environ Res Public Health. 2022 Jun 24;19(13):7763. doi: 10.3390/ijerph19137763 (PMC9265455; doi:10.3390/ijerph19137763)
Supplement: Supplementary file 1 [file ijerph-19-07763-s001.zip › Questionnaire HPV Chinese.pdf]

V. Buzzi 儿童医院
与米兰大学达成协议的高度专业化母婴医院
儿科门诊和儿科急诊科
主任 Gian Vincenzo Zuccotti 教授

INDAGINE SULLA CONSAPEVOLEZZA RIGUARDO ALLA VACCINAZIONE PER HPV
针对 HPV 疫苗了解状况的调查
面向男童和男性青少年父母的问卷

问卷填写者： [ ] 父亲 [ ] 母亲

孩子的年龄：……………….. **父母的年龄：**……………

父母的国籍：……………….. **父母的宗教信仰：** ………………..

父母的学历： [ ] 初中 [ ] 高中 [ ] 大学

1) 你了解 HPV 病毒吗，知道它与哪些疾病有关吗？
[ ] 了解 [ ] 只是听说过 [ ] 不了解

2) 如果了解，是谁告诉你的？
[ ] 儿科医生 [ ] 疫苗中心 [ ] 朋友/亲人

3) 您知道在伦巴第大区，2006 年以后出生的男性可以免费接种疫苗吗？
[ ] 知道 [ ] 不知道

4) 你们希望了解更多相关信息吗？
[ ] 是 [ ] 否

5) 你计划让自己的儿子接种吗？
[ ] 已经接种 [ ] 是 [ ] 否

如果为否，为什么？ ………………………………………………………………………

6) 你有其他儿子/女儿接种了 HPV 疫苗吗？（如果是，请指出性别和年龄）
[ ] 没有 [ ] 有 ……………………………………………………

7) 总的来说，你赞成接种疫苗吗？
[ ] 赞成 [ ] 不赞成 [ ] 不知道

针对孩子年龄超过 15 岁的父母：

a) 你的儿子接种了 HPV 疫苗吗？
[ ] 是 [ ] 否

b) 如果疫苗已包含在免费疫苗接种项目中，你会让你的孩子接种吗？
[ ] 会 [ ] 不会

**Servizio Sociale Professionale Sportello Orientamento Stranieri**	**Traduzione effettuata da: Eurostreet**	**Data 10/11/2021**
