# Supplementary material for: HPV Vaccination in Young Males: A Glimpse of Coverage, Parental Attitude and Need of Additional Information from Lombardy Region, Italy
Source: Int J Environ Res Public Health. 2022 Jun 24;19(13):7763. doi: 10.3390/ijerph19137763 (PMC9265455; doi:10.3390/ijerph19137763)
Supplement: Supplementary file 1 [file ijerph-19-07763-s001.zip › Questionnaire HPV Arabic.pdf]

Ospedale dei Bambini V. Buzzi
Ospedale di alta specializzazione materno-infantile convenzionato con l'Università degli Studi di Milano

Clinica Pediatrica e Pronto Soccorso Pediatrico

Direttore prof. Gian Vincenzo Zuccotti

INDAGINE SULLA CONSAPEVOLEZZA RIGUARDO ALLA VACCINAZIONE PER HPV

استبيان حول الوعي بما يتعلق بالتطعيم ضد فيروس الورم الحليمي البشري

استبيان موجه لأولياء أمور الأطفال والمراهقين الذكور

تم ملء الاستبيان من قبل: [ ] الأب [ ] الأم

عمر الطفل: .... **عمر الوالد:**...............

جنسية الوالد: .... **ديانة الوالد:** ....

التأهيل التعليمي لولي الأمر: [ ] المدرسة الإعدادية [ ] المدرسة الثانوية [ ] شهادة جامعية

1) هل تعرف فيروس الورم الحليمي البشري وهل تعرف الأمراض المرتبطة به؟

[ ] نعم [ ] سمعت عنها فقط [ ] لا

2) إذا كان الجواب نعم ، من أخبركم عنه؟

[ ] طبيب الأطفال [ ] مركز التطعيم [ ] الأصدقاء / الأقارب

3) هل تعلم أنه في منطقة لومباردي يتم تقديم اللقاح مجانًا للذكور المولودين بعد عام 2006؟

[ ] نعم [ ] لا

4) هل ترغب على الحصول على المزيد من المعلومات حول هذا الموضوع؟

[ ] نعم [ ] لا

5) هل ستقوم بتطعيم طفلكم؟

[ ]تم تلقيحه بالفعل [ ] نعم [ ] لا

إذا كان الجواب لا ، فلماذا؟ ....

6) هل لديك أبناء / بنات آخرون تم تطعيمهم ضد فيروس الورم الحليمي البشري؟ (<u>إذا كانت الإجابة نعم، حدد الجنس والعمر</u>)

[ ] لا [ ] نعم ....

7) بشكل عام هل تؤيد التطعيمات؟

[ ] نعم [ ] لا [ ] لا أعرف

<u>لآباء الأطفال> 15 سنة:</u>

أ) هل تم تطعيم طفلك ضد فيروس الورم الحليمي البشري؟

[ ] نعم [ ] لا

ب) إذا كان اللقاح قد تم تصنيفه مع التطعيمات المجانية، فهل ستقوم بتطعيم طفلك؟

[ ] نعم [ ] لا

Servizio Sociale Professionale Sportello Orientamento Stranieri	Traduzione effettuata da: Eurostreet	Data 10/11/2021
